# Growth and characterization of ZnO/ZnTe core/shell nanowire arrays on transparent conducting oxide glass substrates

**DOI:** 10.1186/1556-276X-7-401

**Published:** 2012-07-17

**Authors:** YuWei Lin, Wei-Jen Chen, Jiun You Lu, Yuan Huei Chang, Chi-Te Liang, Yang Fang Chen, Jing-Yu Lu

**Affiliations:** 1 Graduate Institute of Applied Physics, National Taiwan University, Taipei, 10617, Taiwan; 2 Department of Physics, National Taiwan University, Taipei, 10617, Taiwan

**Keywords:** ZnO/ZnTe core/shell nanowire arrays, indium tin oxide, glass substrates

## Abstract

We report the growth and characterization of ZnO/ZnTe core/shell nanowire arrays on indium tin oxide. Coating of the ZnTe layer on well-aligned vertical ZnO nanowires has been demonstrated by scanning electron microscope, tunneling electron microscope, X-ray diffraction pattern, photoluminescence, and transmission studies. The ZnO/ZnTe core/shell nanowire arrays were then used as the active layer and carrier transport medium to fabricate a photovoltaic device. The enhanced photocurrent and faster response observed in ZnO/ZnTe, together with the quenching of the UV emission in the PL spectra, indicate that carrier separation in this structure plays an important role in determining their optical response. The results also indicate that core/shell structures can be made into useful photovoltaic devices.

## Background

ZnO is a material with great potential for a wide variety of practical applications. ZnO has a bandgap of 3.37 eV at room temperature and is emerging as a potential alternative to GaN in optoelectronic application, including piezoelectric transducers, optical waveguides, and transparent conducting oxides [1]. In addition, ZnO has a high exciton binding energy of 60 meV that makes it an ideal candidate for optical devices such as UV light-emitting diodes, UV lasers [2], and UV photodetectors. In the past decade, ZnO nanowire-based photovoltaic devices, such as ZnO single-nanowire photodetectors, which have a small volume, a small active region, and a high internal gain [3]; ZnO p-n junction nanowire photodetectors, which exhibit clear rectifying characteristics and good ultraviolet light absorption [4]; and ZnO nanowire-quantum dot photovoltaic devices which can increase the absorption region with different wavelengths [5], have been studied. Recently, type-II heterojunction core/shell nanowires [6-9] have attracted much attention because the band alignment of these structures can separate the electron and hole into different spatial regions and thus can increase the carrier lifetimes. Vertically aligned core/shell nanowire arrays with these kinds of band structure can find wide applications in optoelectronic devices such as solar cells because they have higher surface-to-volume ratio, better light-trapping effect, and longer carrier lifetime as compared to the planar structures [10].

Amongst the type-II core/shell structures, the ZnO-based nanowire array is of particular interest because ZnO has excellent chemical stability, high surface-to-volume ratio, high refractive index, is nontoxic, and is friendly to the environment. As a result, interesting results have been obtained in ZnO/ZnSe [6], ZnO/ZnS [7], and ZnO/CdTe [8,9] nanowire arrays. On the other hand, the ZnO/ZnTe nanowire array is expected to be an ideal system for photovoltaic applications [10]. First, as-grown ZnTe is usually a p-type material and as-grown ZnO is usually n-type; therefore, n-ZnO/p-ZnTe heterostructure nanowires such as nano-p-n junctions and photodiodes may be readily fabricated. Second, like ZnO, ZnTe is nontoxic and is thus environment-friendly. Most importantly, the bandgap of bulk ZnTe is 2.34 eV, which is considerably smaller than that of ZnO and can greatly enhance absorption in the visible spectrum. Given the present intense interest in core/shell photodiodes [11] and one-dimensional nanostructures [12], it is therefore highly desirable to prepare and investigate ZnO/ZnTe nanowire arrays. However, to date, there seems to be a dearth of study of ZnO/ZnTe core/shell nanowire arrays, and it is the purpose of this letter to report the successful growth of such a structure. It is found that ZnTe can be coated on ZnO nanowires as supported by scanning electron microscope (SEM), transmission electron microscope (TEM), X-ray diffraction (XRD) pattern, photoluminescence (PL), and transmission studies. We also show that our ZnO/ZnTe core/shell array can have good optical properties and may find applications as a photovoltaic device.

## Methods

We now describe the fabrication process of our ZnO/ZnTe core/shell array. Indium tin oxide (ITO) glass substrates, with a thickness of 135 nm and an electrical resistivity of 15 Ω/sq, were chosen to be used as transparent conducting oxide glass substrates because of their optical transparency and high electrical conductivity. The ITO glass substrate was then cut into 10 mm × 20 mm pieces. We note that it is important to clean the substrate thoroughly so as to achieve successful growth of our devices. To this end, the ITO glass substrate was cleaned ultrasonically in acetone, ethanol, and deionized water. Finally, the substrates were dried and cleaned by blowing dry nitrogen gas. High-density ZnO nanowire arrays with low defect concentrations can now be directly grown on ITO glass substrates under catalyst-free and low-temperature conditions by chemical vapor deposition (CVD). About 0.5 g of zinc powders was put on the alumina boat, and the clean ITO glass substrate was placed at 2 cm downstream to zinc powders on the same alumina boat. Then, the alumina boat was inserted into the center of the quartz tube in the furnace to grow ZnO nanowire arrays. The furnace was heated under a constant flow of 37 sccm Ar and 5 sccm O_2_ to 600°C for the growth of ZnO nanowire arrays. This reaction at 600°C reacted for 40 min, and then the furnace was turned off. The furnace was allowed to cool down to room temperature. In this way, ZnO nanowire arrays were successfully grown on the ITO glass substrate [13]. The ZnO nanowire arrays on the ITO glass substrate were transferred into the metal oxide chemical vapor deposition (MOCVD) chamber to deposit the ZnTe shell for 700 s at 550°C and 760 Torr. Nitrogen was the carrier gas for the precursor combination dimethylzinc (DMZn) and dimethyl telluride (DMTe). It was essential to control the flow rate precisely because the ZnTe shell was just only tens of nanometer. The optimal flow rate of DMZn and DMTe was chosen to be 1:2 to flow through the MOCVD chamber by MFC [14].

## Results and discussion

**Figure 1:**
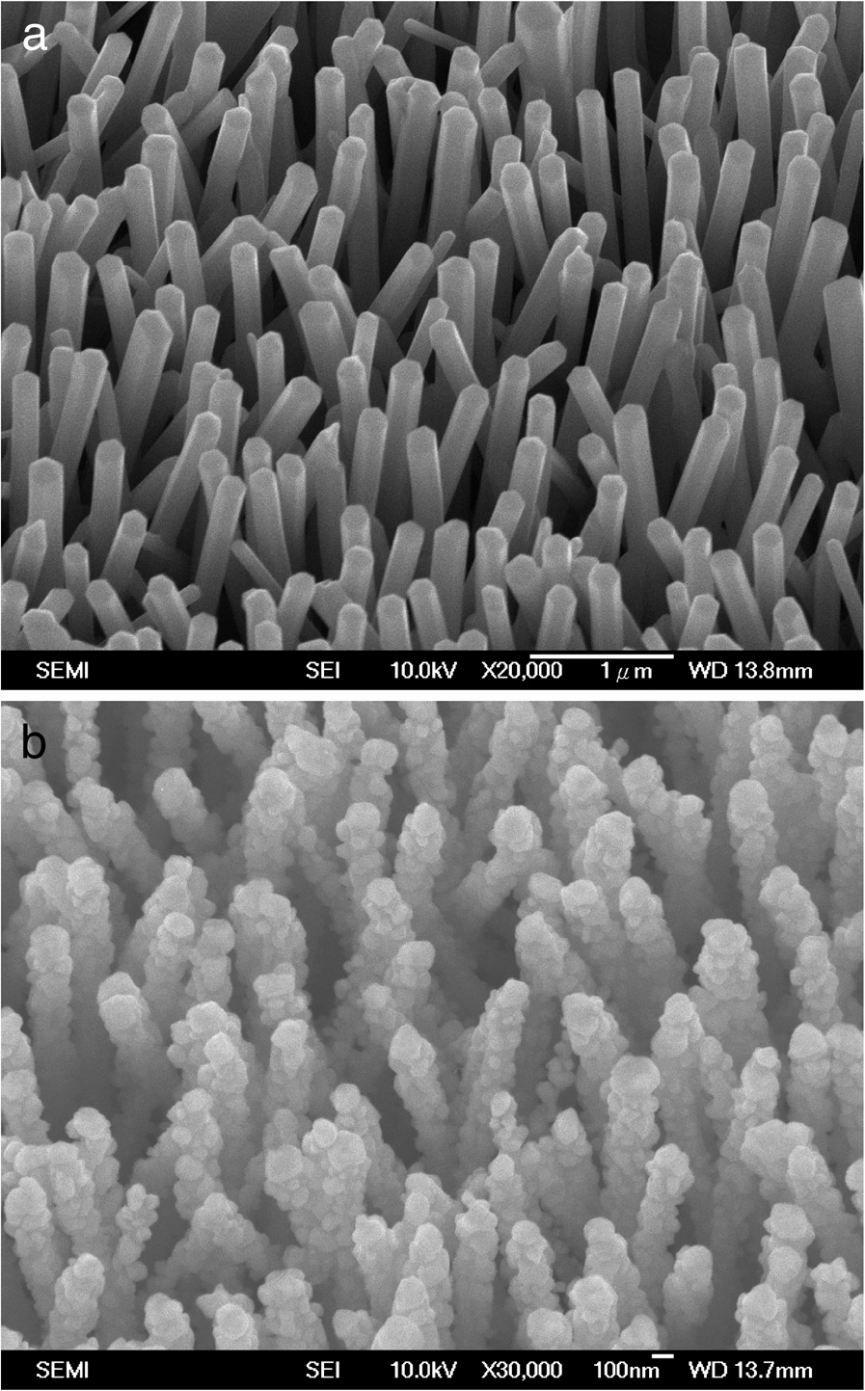
** SEM images.** (**a**) SEM image of the well-aligned ZnO nanowire arrays. (**b**) SEM image of the well-aligned ZnO/ZnTe core/shell nanowire arrays with a depositing time of 700 s.

We now describe our main experimental results. Figure 1a shows the SEM image of as-synthesized ZnO nanowire arrays. As shown in this figure, the ZnO nanowire arrays were grown perpendicularly on the ITO glass substrate with an average length of approximately 2 μm and diameters in the range of 200 to 250 nm. Figure 1b shows the SEM image of ZnO/ZnTe core/shell nanowire arrays. The noticeably increased diameters and rough surfaces clearly demonstrate that ZnTe was successfully deposited over the ZnO nanowires.

**Figure 2:**
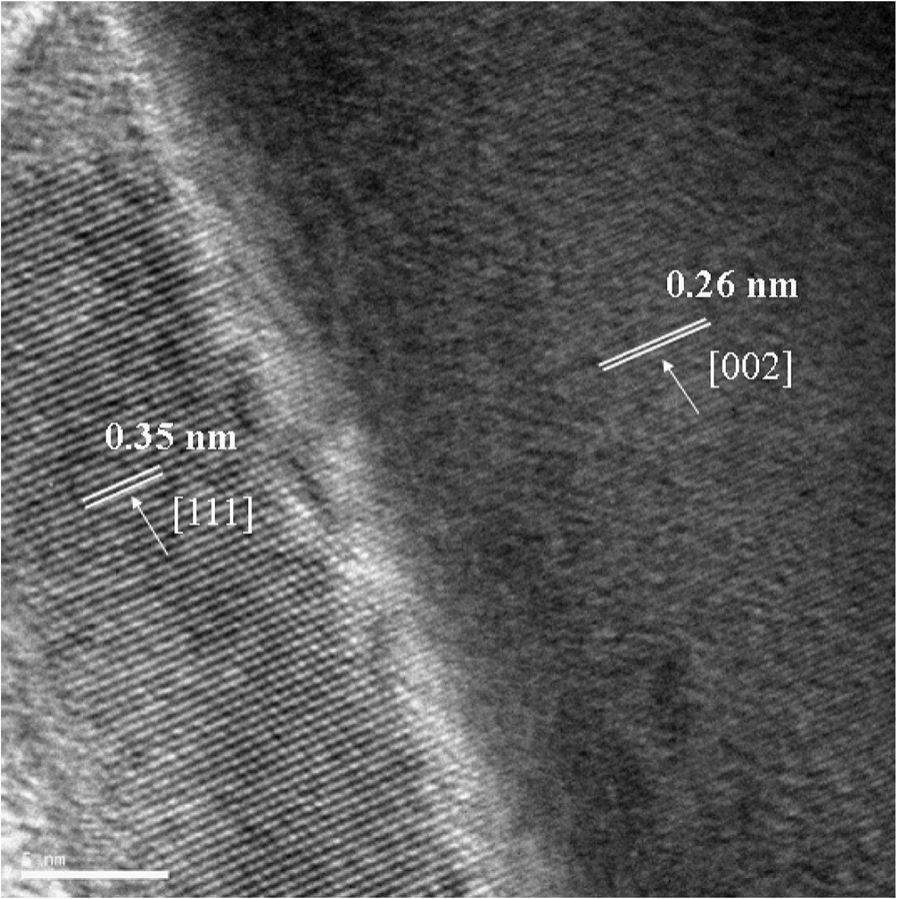
High-magnification TEM image near the interface between ZnO [002] and ZnTe [111].

Figure 2 shows a high-magnification TEM image of a ZnO/ZnTe core/shell nanowire, revealing that the ZnTe shell was deposited directly in the radial direction from the surface of the ZnO core. This result shows the lattice constants of the bare ZnO core and the ZnTe shell, indicating that the interplanar spacing of 0.26 nm corresponds to the [002] lattice plane of wurtzite ZnO and the interplanar spacing of 0.35 nm corresponds to the [111] lattice plane of zincblende ZnTe.

**Figure 3:**
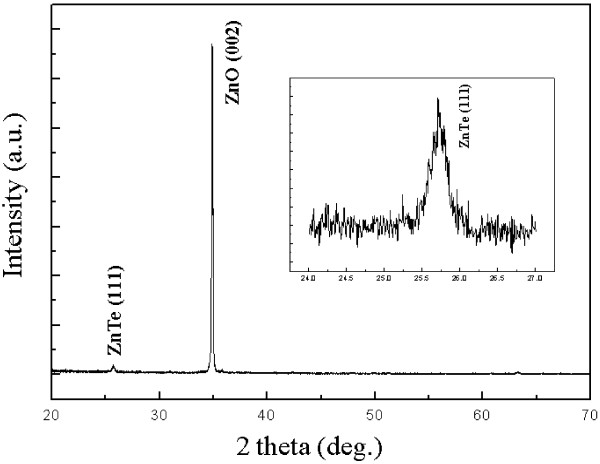
** XRD patterns of ZnO/ZnTe core/shell nanowire arrays.** The inset shows clearly the ZnTe diffraction peak at 25.71°.

**Figure 4:**
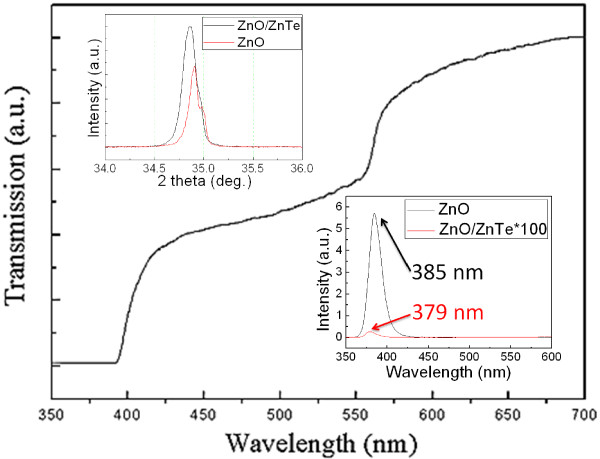
** Transmission spectra of ZnO/ZnTe core/shell nanowire arrays.** The top inset shows the XRD data, revealing a small shift of the ZnO (002) diffraction peak after being deposited with the ZnTe layer. The bottom inset shows room-temperature photoluminescence spectra of ZnO and ZnO/ZnTe nanowire arrays.

To further study the crystal quality of our device, we show the XRD patterns of the ZnO/ZnTe core/shell nanowire array in Figure 3. The strong hexagonal ZnO (002) diffraction peak is observed in the XRD patterns. Moreover, another diffraction peak is observed in addition to the diffraction peak from the hexagonal ZnO nanowire arrays after the deposition of ZnTe. This diffraction peak at 25.71°, shown clearly in the inset of Figure 3, is attributed to the diffraction from the (111) plane of ZnTe with a zincblende structure. The intensity of the ZnTe (111) diffraction peak is weak because the ZnTe layer is very thin. Compared with that of the bare ZnO nanowire arrays, the position of the ZnO (002) diffraction peak after being deposited with the ZnTe layer shows a small shift to the smaller angle side in the top inset of Figure 4, which indicates that the ZnO lattice is enlarged. The small shift may be attributed to the lattice expansion induced by the ZnTe shell growth.

The bottom inset of Figure 4 shows the PL spectra of ZnO and ZnO/ZnTe core/shell nanowire arrays measured at room temperature. The PL peak near the band edge of ZnO is found to be very strong with negligible emission peaks associated with deep-level defects. Compared with that of the bare ZnO nanowire arrays, the peak position of the UV emission in ZnO/ZnTe core/shell nanowire arrays shows a small blueshift, and the intensity is reduced by a factor of 850. This result also indicates that the charge carrier separation driven by the type-II band alignment between ZnO and ZnTe serves as the major contributor to the quenching of the PL peak at 384 nm near the band edge of ZnO, while interfacial recombination, depletion, and photon blocking have little contributions.

Figure 4 shows the transmission spectra of ZnO/ZnTe core/shell nanowire arrays. There are two abrupt drops near 390 and 550 nm in the transmission curve, corresponding well to the bandgap of ZnO and ZnTe, respectively. The transmission intensity of ZnO/ZnTe core/shell nanowire arrays not only increases at 390 and 550 nm but also increases gradually in other regions. The component in other regions can raise from a spatially indirect or an interfacial transition, coupling a hole state in the ZnTe shell with an electron state in the ZnO core.

**Figure 5:**
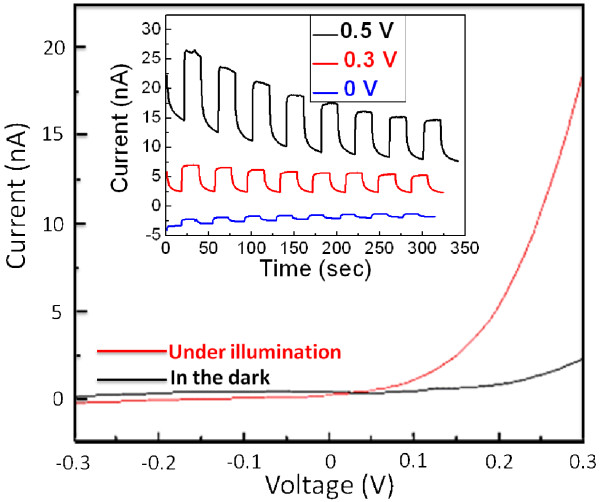
**I-V curves for ZnO/ZnTe core/shell nanowire arrays measured in the dark and under light illumination.** The inset shows time-dependent photocurrent of ZnO/ZnTe core/shell nanowire arrays under different external biases.

Finally, we demonstrate that our device can be utilized as a photovoltaic device. To this end, the ZnO/ZnTe core/shell nanowire array was first coated with a thin layer of gold by sputtering at 20 mA for 4 min. Then, a part of the substrate was etched with nitric acid which almost did not etch the ITO film. Therefore, a part of the ITO film was uncovered to serve as the electrode, and the other electrode was a layer of gold. Besides, all sides of the substrate were also etched with nitric acid in order to avoid the leakage current. Eventually, the ZnO/ZnTe core/shell nanowire arrays are used as the active layer and carrier transport medium. Figure 5 shows the current versus voltage (I-V) curves for the ZnO/ZnTe nanowire arrays measured both in the dark and under light illumination through a xenon arc lamp with an estimated power density of about 40 mW cm^−2^. In the dark, the I-V curve shows typical rectifying behavior, with a weak current of approximately 0.7 nA at a voltage of 0.2 V. The larger photocurrent of approximately 5.2 nA is observed with 0.2-V bias potential under light illumination. This represents more than sevenfold increase in the current density over the measurement performed in the dark. The photoresponse time of ZnO/ZnTe core/shell nanowire arrays shown in the inset of Figure 5 is less than 0.2 s which means that steady and prompt photocurrent generate can be obtained during on and off cycles of illumination. Besides, the photocurrent is observed to increase with the bias. The enhanced photocurrent and faster response observed in ZnO/ZnTe can indicate the realization of the key feature of the type-II heterostructure separating the electrons and holes.

## Conclusions

In summary, well-aligned ZnO/ZnTe core/shell nanowire arrays were successfully fabricated on transparent conducting oxide glass substrates by CVD and MOCVD. The structures' properties were investigated in detail by SEM, TEM, and XRD studies; the results showed the core/shell structure that the ZnTe shell deposited directly in the radial direction from the surface of the ZnO nanowire. The ZnO core was consisted of the (002) plane of wurtzite structure; the ZnTe shell was consisted of the (111) plane of zincblende structure. The optical properties were investigated by PL and transmission studies. The results showed that the ZnO/ZnTe core/shell nanowires have desirable optical properties. The ZnO/ZnTe core/shell nanowire arrays were then used as the active layer and carrier transport medium to fabricate a photovoltaic device. The enhanced photocurrent and faster response observed in ZnO/ZnTe, together with the quenching of the UV emission in the PL spectra, indicate that carrier separation in this structure plays an important role in determining their optical response. The results also indicate that core/shell structures can be made into useful photovoltaic devices.

## Abbreviations

CVD, chemical vapor deposition; ITO, indium tin oxide; MOCVD, metal oxide chemical vapor deposition; PL, photoluminescence; SEM, scanning electron microscope; TEM, transmission electron microscope; XRD, X-ray diffraction.

## Competing interests

The authors declare that they have no competing interests.

## Authors’ contributions

YWL and WJC performed the measurements. JYL and JYL performed the SEM and TEM measurements. YHC and YFC coordinated the projects. YWL, YHC, and CTL drafted the manuscript. All authors read and agreed the final version of the manuscript.

## Authors’ information

YWL obtained his M.Sc. degree at National Taiwan University (NTU). WJC obtained his M.Sc. degree at NTU and is currently a Ph.D. student working at the Department of Physics, NTU. JYL obtained his Ph.D. degree at NTU and is currently an engineer working for TSMC, Taiwan. YHC obtained his B.Sc. degree at National Tsing Hua University, Taiwan and his Ph.D. degree at the University at Buffalo, USA, and is currently the Director of the Science and Technology Division, Taipei Economic and Cultural Center in India and a professor of Physics, NTU. CTL obtained his B.Sc. degree at NTU in 1990 and his Ph.D. degree in Physics at Cambridge University, UK in 1996 and is currently a professor of Physics at NTU. YFC obtained his B.Sc. degree at National Tsing Hua University, Taiwan and his Ph.D. degree at Purdue University, USA, and is currently a chair professor at NTU. JYL obtained his B.Sc. degree at Fu Jen Catholic University, Taiwan, and his M.Sc. degree at Tamkang University, Taiwan, and is currently a technician in charge of the TEM facility, NTU.
